# A Genetic Basis for Mechanosensory Traits in Humans

**DOI:** 10.1371/journal.pbio.1001318

**Published:** 2012-05-01

**Authors:** Henning Frenzel, Jörg Bohlender, Katrin Pinsker, Bärbel Wohlleben, Jens Tank, Stefan G. Lechner, Daniela Schiska, Teresa Jaijo, Franz Rüschendorf, Kathrin Saar, Jens Jordan, José M. Millán, Manfred Gross, Gary R. Lewin

**Affiliations:** 1 Department of Neuroscience, Max-Delbrück Center for Molecular Medicine, Berlin-Buch, Germany; 2 Department of Audiology and Phoniatrics, Charité Universitätsmedizin, Berlin, Germany; 3 Institute of Clinical Pharmacology, Hannover Medical School, Hannover, Germany; 4 Genetics Unit, Hospital Universitario La Fe, and CIBERER, Valencia, Spain; 5 Experimental Genetics of Cardiovascular Disease, Max-Delbrück Center for Molecular Medicine, Berlin-Buch, Germany; The Wellcome Trust Centre for Human Genetics, University of Oxford, United Kingdom

## Abstract

In all vertebrates hearing and touch represent two distinct sensory systems that both rely on the transformation of mechanical force into electrical signals. There is an extensive literature describing single gene mutations in humans that cause hearing impairment, but there are essentially none for touch. Here we first asked if touch sensitivity is a heritable trait and second whether there are common genes that influence different mechanosensory senses like hearing and touch in humans. Using a classical twin study design we demonstrate that touch sensitivity and touch acuity are highly heritable traits. Quantitative phenotypic measures of different mechanosensory systems revealed significant correlations between touch and hearing acuity in a healthy human population. Thus mutations in genes causing deafness genes could conceivably negatively influence touch sensitivity. In agreement with this hypothesis we found that a proportion of a cohort of congenitally deaf young adults display significantly impaired measures of touch sensitivity compared to controls. In contrast, blind individuals showed enhanced, not diminished touch acuity. Finally, by examining a cohort of patients with Usher syndrome, a genetically well-characterized deaf-blindness syndrome, we could show that recessive pathogenic mutations in the *USH2A* gene influence touch acuity. Control Usher syndrome cohorts lacking demonstrable pathogenic *USH2A* mutations showed no impairment in touch acuity. Our study thus provides comprehensive evidence that there are common genetic elements that contribute to touch and hearing and has identified one of these genes as *USH2A*.

## Abstract 2

Hearing and touch are genetically related, and people with excellent hearing are more likely to have a fine sense of touch and vice versa.

## Author Summary

In humans many genes have been identified that cause deafness when mutated, but no equivalent genes have been identified that are required for touch. Here, we asked whether genes that influence hearing can also influence touch. Using identical and non-identical human twins it was possible to show that touch performance is substantially influenced by genes. Furthermore, people who have excellent hearing are more likely to have a fine sense of touch and vice versa. Interestingly, people who suffer from congenital deafness have a higher chance of having poor touch performance. In a genetically defined form of human deafness, Usher syndrome type II, a single mutated gene was identified that also impairs touch. Touch and hearing are thus intricately intertwined and there may be other touch/hearing genes waiting to be discovered.

## Introduction

All animals are equipped with a range of specialized sensory cells whose prime function is to detect mechanical force. The most familiar of these sensory systems are hearing and touch, but mechanosensory cells also detect important stimuli that are not consciously perceived, for example, changes in blood pressure. We reasoned that since the prime function of different sensory cells is to detect mechanical force they may utilize a common set of mechanosensory proteins for this function. According to this hypothesis genetic variation affecting the function of mechanosensory proteins would be predicted to quantitatively change more than one mechanosensory trait. Genetics has been very successfully used to characterize new molecules that are essential for human hearing [1]. There are over 60 known genes linked to sensorineural hearing loss, and a similar number of loci linked to hearing impairment exist for which the underlying genetic defect has not been identified (for an updated list see http://hereditaryhearingloss.org). Non-syndromic sensorineural hearing loss is commonly caused by single gene mutations, which primarily affect the function of the sensory hair cells that detect movement of the basilar membrane induced by sound. Sensorineural deafness often manifests from birth and some of the responsible genes encode components of the mechanotransduction apparatus of the hair cell that transforms mechanical force into electrical signals [2]–[4].

In contrast to hearing, virtually nothing is known about the genetics of touch. Indeed there are, to our knowledge, no reported cases of non-syndromic reduced or absent touch sensitivity present from birth in humans. Impaired detection of high frequency vibration (>80 Hz) in humans was recently shown to be associated with pathogenic mutations in the transcription factor c-Maf (MIM:177075, MIM refers to the OMIM database) and may be due to a failure in the development of specific mechanoreceptors associated with Pacinian corpuscles [5]. In contrast, congenital complete insensitivity to pain has been recognized for many years [6] and there are now a small group of genes (*NTRK1*; MIM:191315, *NGFB*; MIM:162030, and *SCN9A*; MIM:603415), mutation of which is known to cause this condition [7]–[10]. Impaired touch sensitivity has been described as one symptom of several severe inherited or acquired neurological disorders ranging from large fiber neuropathy to Charcot-Marie tooth disease [11],[12], however such neurological diseases are often associated with structural changes in the peripheral nervous system. The peripheral sensory nervous system consists of primary sensory neurons located in the cranial and dorsal root ganglia (DRG) and these are the most numerous sensory cells of the body. Thus virtually every somatic tissue of the body, skin, muscle, and visceral organs is innervated by the axons of sensory neurons, which can form mechanosensitive endings in these tissues. The skin represents the largest of our sensory organs and is innervated by a variety of sensory neuron types that can be characterized as low threshold mechanoreceptors [13]. Very little is known about the molecular basis of mechanotransduction in somatic mechanoreceptors, but transduction in these neurons may be accomplished by a multi-protein complex similar to that described for touch receptor neurons (TRNs) in the nematode worm *C. elegans* [4]–[6]. We have previously shown that STOML3 (Swissprot Q6PE84) is required for normal touch-driven behavior in the mouse, and STOML3 is a membrane protein that is required for the function of mechanosensitive ion channels in DRG neurons [4]. But evidence that *Stoml3* mutations are causative for impairments in human touch is so far lacking. But assuming that touch sensitivity is a complex genetic trait, it should be possible to detect a heritable component in the normal variation of touch-related traits, as has been shown before for other sensory traits, such as pain sensitivity and hearing [14],[15].

Here we show that there is a significant genetic component to touch sensitivity in humans by determining the heritability of touch traits, assessed by quantitative sensory testing, in a classical twin study. In accordance with our hypothesis that there are common genetic factors underlying different mechanosensitive systems, we found that quantitative measures of mechanosensory traits—that is, touch acuity, hearing acuity, and baroreflex function—are positively correlated with each other in a healthy human population. We also examined a cohort of people suffering from congenital hearing loss and found touch sensitivity to be poorer in these individuals compared to a control cohort. To investigate the role of single sensorineural deafness genes in cutaneous touch sensitivity, we assessed touch acuity and sensitivity in people suffering from Usher syndrome. We found touch sensitivity to be impaired in a cohort of individuals carrying pathogenic mutations in the *USH2A* gene (MIM:608400), but not in other cases of Usher syndrome. Our study thus provides comprehensive evidence that there are common genetic elements that contribute to touch and hearing and has identified one of these genes as *USH2A*.

## Results

In this study we employed a range of quantitative tests to assess sensory function in a large cohort of volunteers (518 individuals) (summarized in Table 1). Our main aim was to assess mechanosensory-related phenotypic traits, which included two measures of touch sensitivity: a grating orientation task, which assesses the participants' finger tip touch acuity in millimeters, and a vibration detection test, which measures the vibration detection threshold (VDT) for a sinusoidal vibratory stimulus delivered to the finger at 125 Hz. Two aspects of hearing were examined: the psychophysically determined perception threshold for a series of pure tones and click evoked otoacoustic emissions (EOAE). Click evoked otoacoustic emissions were analyzed for the reproducibility of the evoked signal and also for the strength of the evoked signal itself (in dB). Mechanosensory systems are also required for sub-conscious autonomic reflexes like the baroreceptor reflex, in which pressure changes in the large arteries are detected and influence beat-to-beat heart rate in resting participants. We measured the vascular baroreflex with the sequence technique in resting participants [16]. In addition to the sequence slope, the number of baroreflex sequences over a period of 5 min (baroreflex sequence frequency) was determined. All the phenotypic tests described above are direct or indirect measures of mechanosensory function, and so we also included tests of temperature sensation as a control. Four different temperature sensitivity traits were investigated: cold and warmth detection thresholds as well as heat and cold pain detection thresholds. All of these phenotypic measures were employed on volunteers recruited for the first part of this study, which was a classical twin study using mono- and dizygotic twins to estimate heritability values for the investigated traits (see Table 1, Table S3). It is important to know the reproducibility or reliability of such measurements, especially when used in twin studies to estimate heritability of a trait. We thus directly measured test-retest reliability for the touch sensitivity assays employed, and the intra-class correlation coefficients for the two tests performed on the same individuals at an interval of several weeks were 0.90 and 0.61 for VDT and acuity, respectively (*n* = 17). The other tests employed in this study were not analyzed by us for test-retest reliability, but data showing good reliability for pure tone audiometry [17], evoked otoacoustic emissions [18], baroreflex sensitivity [19], warm and cold perception [20], and heat and cold pain threshold [14] have been published.

**Table 1:** Summary of psychophysical and physiological tests carried out in different cohorts.

	Tactile Acuity	Vibrotactile Sensitivity	Hearing Acuity	Otoacoustic Emissions	Baroreflex Function	Temperature Sensitivity	Heat Pain Threshold
Twins	191	187	176	146	176	190	188
Additional controls	151	99	—	42	—	—	—
Blind	57	18	—	—	—	—	—
Congenitally hearing impaired	39	29	—	—	—	—	—
Usher syndrome affected	65	61	—	—	19	51	50
Total	503	394	176	188	195	241	238

Subsets of the complete sensory test battery described above, mostly measuring touch sensitivity, were employed in three follow-up cohorts. Two cohorts were designed to assess the influence of congenital hearing loss or blindness on touch phenotypes. The third cohort was of a group of patients with clinically characterized Usher syndrome, a deaf-blindness syndrome that has been associated with mutations in a small group of genes that are important for hair cell function; for some Usher patients in this study, the mutation was known.

### Age and Sex

In general it has been noted that sensory performance decreases with age and is also influenced by sex [21]–[24]. Each of the phenotypes that we measured exhibited some variability that might be partially accounted for by the age of the participant or his or her sex. Indeed, the participants' performance in all tests, with the exception of baroreflex sequence frequency, showed significant deterioration with age (Figures S1, S2, S3, S4 and Table S1). In order to more reliably compare phenotypic data from participants who ranged in age from 14–68 years, we determined the best mathematical fit for each set of phenotypic data (Table S1). The mean age of the entire control cohort was 27.0±0.7 years, and the median age was 24 years (*n* = 352). In most cases the changes in the sensory trait was best fit by a second order polynomial function, in two cases the age dependence was best described by a linear fit (baroreflex sequence frequency and heat pain threshold), and in one case (baroreflex slope) the data were best fit by an exponential decay function. Here with increasing age the baroreflex sequence slope tends to become shallower, which reflects weaker engagement of the reflex by changes in blood pressure. For subsequent analyses the phenotypes measured were normalized on the basis of the mathematical fits to the mean age of the entire cohort.

Of the 13 sensory traits measured, significant sex differences were found for six traits, and in every case women performed better than men when age-matched cohorts were compared (Figure S5). The sensory performance of women was significantly better than that of men for tactile acuity, otoacoustic emission reproducibility and strength, baroreflex sequence frequency, as well as cold and warmth detection thresholds (Figure S5, Table S2). The absolute magnitude of the sex differences measured in the sensory traits was relatively modest compared to the influence of age. About the same number of woman as men were included in the entire study; 223 males (43%) and 295 females (57%), but the recruitment for the twin study was biased towards females (see below).

### Heritability of Sensory Traits

The heritability *h^2^* of the investigated sensory traits—that is, the component of the variation of the respective trait that can be accounted for by additive genetic effects—was determined in a classical twin study. Of the 100 twin pairs who participated in the study, 38 were monozygotic female pairs, 28 monozygotic male pairs, 25 dizygotic female twin pairs, and 9 dizygotic male twin pairs; no mixed sex twins were recruited. The ages of the twins in our cohort ranged from 18 to 68 years, with a mean age of 29.7±1.14. Zygosity was confirmed using a chip-based method with 9,080 informative autosomal SNPs derived from the so-called Immunochip [25],[26]. Most twin pairs were tested for all parameters, but in some cases this was not possible for organizational reasons. Heritability values were estimated by structural equation modeling; the model employed was an ACE model, in which the variance of the trait is determined by additive genetic effects A, the common environment C, and the unique environment E [27]. The values for the twins were previously corrected for age effects (see Table S1) before being subjected to structural equation modeling. Correcting for both age and sex effects before modeling or treating age and sex as fixed effects in the structural equation modeling did not lead to major changes in heritability estimates (unpublished data).

#### Heritability of touch traits

**Figure 1:**
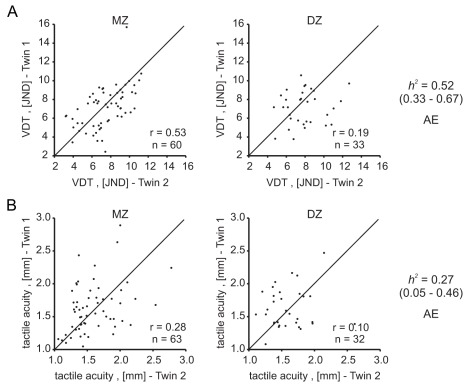
Cross-twin correlations and heritability estimates of touch sensitivity traits. For both vibration detection threshold (A) and tactile acuity (B) cross-twin correlations were higher in monozygotic (MZ) than in dizygotic (DZ) twins and significant heritability values could be estimated. *r*, intra-class correlation; *h^2^*, heritability estimate; 95% confidence interval in brackets; AE, preferred model used to estimate heritability.

For both touch sensitivity traits, vibration detection threshold, and tactile acuity, cross-twin correlations were more than twice as strong in monozygotic twin pairs compared to dizygotic twin pairs (Figure 1, Table S3). Of the two touch traits the most robust correlations were found for vibration detection threshold. Significant heritability values could be estimated for both traits. The heritability estimate for tactile acuity was lower at 0.27 (95% CI = 0.05–0.46), whereas the estimate for heritability of the vibration detection threshold was high at 0.52 (95% CI = 0.33–0.67). For both touch sensitivity traits the AE model best described the data and was used to estimate the heritability.

#### Heritability of hearing traits

**Figure 2:**
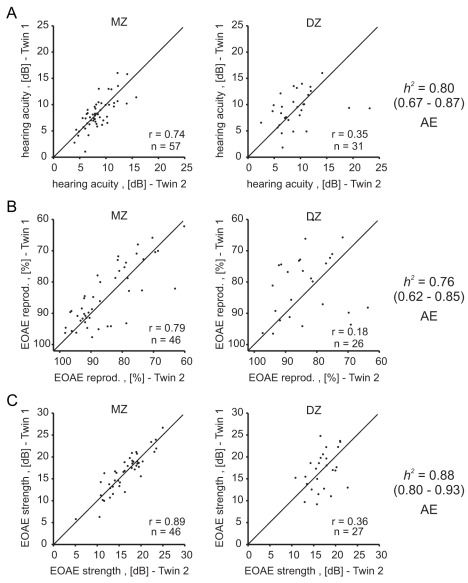
Cross-twin correlations and heritability estimates of hearing traits. For all three hearing traits—pure tone thresholds (A), otoacoustic emission reproducibility (B), and otoacoustic emission strength (C)—cross-twin correlations were higher in MZ than in DZ twins, and very high heritability values could be estimated. *r*, intra-class correlation; *h^2^*, heritability estimate; 95% confidence interval in brackets; AE, preferred model used to estimate heritability.

For all three hearing traits the cross-twin correlations were more than twice as strong in the monozygotic twin pairs compared to dizygotic twin pairs, with high overall correlations (Figure 2, Table S3). The heritability estimates were exceptionally high, 0.80 (95% CI = 0.67–0.87) for hearing acuity, 0.76 (95% CI = 0.62–0.85) for EOAE reproducibility, and even 0.88 (95% CI = 0.80–0.93) for EOAE strength. For all three hearing traits the AE model best described the data and was used to estimate the heritability.

#### Heritability of baroreflex traits

**Figure 3:**
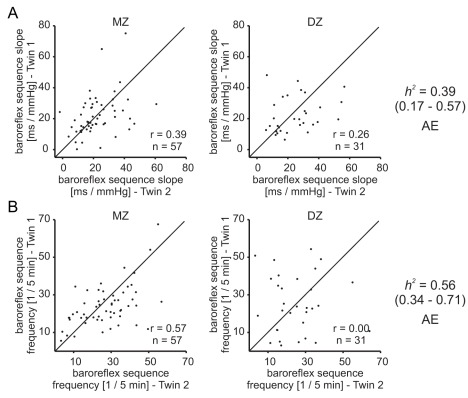
Cross-twin correlations and heritability estimates of baroreflex traits. For both baroreflex traits—baroreflex sequence slope (A) and baroreflex sequence frequency (B)—the cross-twin correlations were higher in MZ than in DZ twins. Significant heritability estimates could be calculated. *r*, intra-class correlation; *h^2^*, heritability estimate; 95% confidence interval in brackets; AE, preferred model used to estimate heritability.

The cross-twin correlations for the baroreflex traits were higher in the monozygotic twin pairs than in the dizygotic twin pairs (Figure 3, Table S3). Significant heritability estimates could be calculated using the AE model. The heritability estimate for the baroreflex slope was 0.39 (95% CI = 0.17–0.57) and higher for baroreflex sequence frequency at 0.56 (95% CI = 0.34–0.71).

#### Heritability of temperature sensation

**Figure 4:**
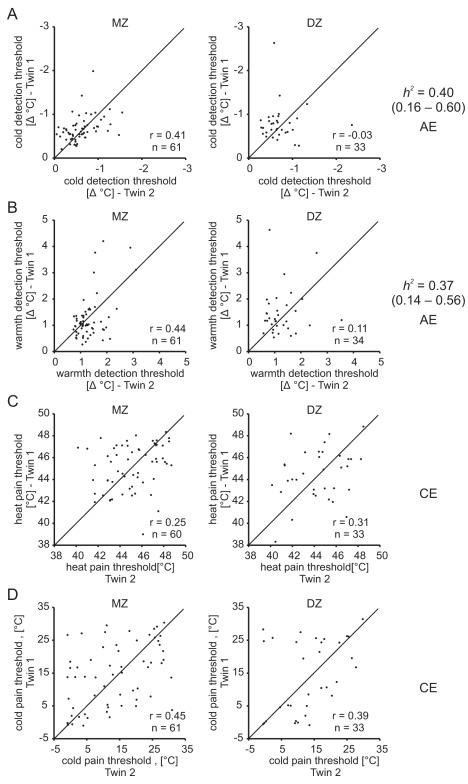
Cross-twin correlations and heritability estimates (where applicable) of temperature sensitivity traits. For all traits except the cold pain threshold, the cross-twin correlations were higher in MZ than in DZ twins. Significant heritability estimates could be calculated for cold (A) and warmth (B) detection thresholds, whereas for the heat (C) and cold (D) pain thresholds, the CE model, not containing a heritable component, was preferred. *r*, intra-class correlation; *h^2^*, heritability estimate; 95% confidence interval in brackets; AE/CE, preferred model used to estimate heritability.

The cross-twin correlations for all temperature sensitivity traits, except cold pain threshold, were higher in the monozygotic twin pairs than in the dizygotic twin pairs (Figure 4, Table S3). Significant heritability estimates could be calculated for cold and warmth detection thresholds using the AE model. The estimates for warmth and cold detection were 0.40 (95% CI = 0.16–0.60) and 0.37 (95% CI = 0.14–0.56), respectively. A heritability value was not calculated for heat and cold pain thresholds since the data were best fit with a CE model, which does not include a genetic component.

### Good Hearing, Good Touch?

**Figure 5:**
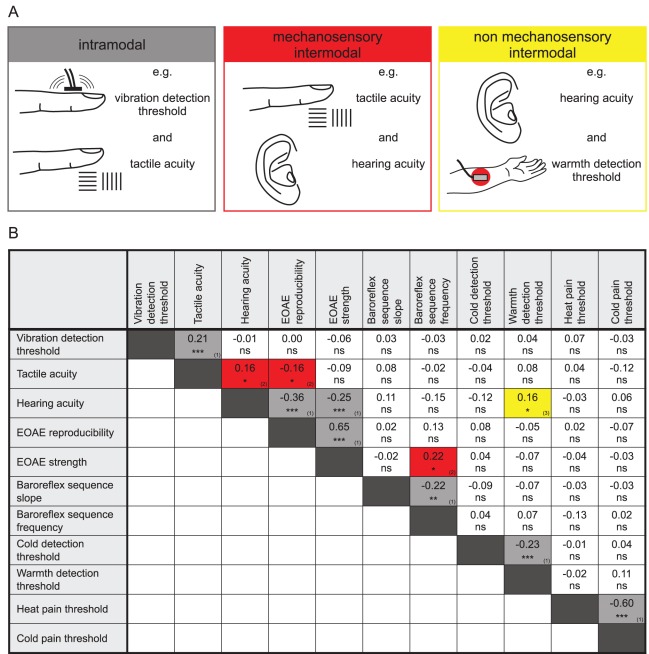
Cross-correlations between the investigated sensory traits. Three different types of intramodal and intermodal correlations were distinguished (A). Three mechanosensory and one non-mechanosensory intermodal correlation were detected (B). ^ns^, not significant; * *p*<0.05; ** *p*<0.01; *** *p*<0.001. False discovery rates for *p* value cutoff at 0.05 for (1) intramodal correlations: 0.004, (2) mechanosensory intermodal correlations: 0.14, and (3) non-mechanosensory intermodal correlations: 0.83. Values were corrected for age before analysis.

If there are common genetic variants that can influence all mechanosensory traits, we would expect to see a correlation between different mechanosensory traits (mechanosensory intermodal comparison). As a prerequisite for an intermodal correlation, one should observe strong correlations between different measures of one sensory system, such as between tactile acuity and vibration detection threshold (intramodal comparison). This was indeed the case as all measures of one sensory system showed a significant correlation with each other, although in many cases this correlation coefficient (*r*) was surprisingly low, but ranged from *r* = 0.21 for VDT and tactile acuity to *r* = 0.65 for otoacoustic emission strength against otoacoustic emission reproducibility (Figure 5). Notable was the fact that strong and significant correlations were found between cold detection threshold and warmth detection threshold (*r* = −0.23) as well as between cold pain and heat pain threshold (*r* = −0.60). We then reasoned that if a group of gene variants positively influences one trait like touch, they may also positively influence another mechanosensory trait in which they also play a functional role (e.g., hearing). Put simply the question is, If someone has good hearing, are they also more likely to have good touch sensitivity? We made such comparisons for all the phenotypic parameters measured from our twin cohort as well as from an additional cohort of healthy individuals. Significant intermodal correlations between mechanosensory traits were detected between tactile acuity and hearing acuity with *r* = 0.16 (*p*<0.05) and tactile acuity and EOAE reproducibility with *r* = −0.16 (*p*<0.05). Additionally, we noted a significant intermodal correlation between EOAE strength and baroreflex sequence frequency (Figure 5). There was just one case of a significant correlation between a mechanosensory and non-mechanosensory trait (i.e., between hearing acuity and warmth detection threshold, with *r* = 0.16, *p*<0.05) (Figure 5). It is possible that because women often perform better than males in some sensory tests (e.g., EOAE strength and warmth detection) (see above), then intermodal correlations may be strengthened because of the presence of females in the cohort. In order to test this idea in cases where a significant sex difference was found, we made a mathematical correction of the raw data so that the male value was corrected to be equivalent to the female value. This was done by making the *corrected male value = (mean female value−mean male value)+measured male value*. After the raw data were adjusted in this way and intermodal correlations tested again, the only statistically significant correlation that remained was between tactile acuity and hearing acuity with *r* = 0.15 (*p*<0.05) (Figure S6). The fact that fewer significant correlations are found between mechanosensory traits after a correction for the person's sex does not necessarily mean that the lost intermodal correlations are not due to common genetic determinants. The evidence thus suggests that there may be genetic factors that have a common influence on more than one mechanosensory trait. The presence of statistically significant intermodal and intramodal correlations between the different sensory traits was not corrected for multiple testing. However, for the analysis shown in Figure 5 we calculated a false discovery rate (FDR) based on the *p* values obtained for intramodal, intermodal mechanosensory, and intermodal non-mechanosensory correlations [28]. This calculation revealed a very low FDR for intramodal correlations (0.004) but also indicated that the FDR for intermodal non-mechanosensory correlations was much higher (0.83) than for intermodal mechanosensory correlations (0.14) (see Figure 5). Thus in order to more rigorously test the hypothesis that common genes influence both hearing and touch sensitivity, we chose to study the touch sensitivity of individuals likely to carry serious genetic lesion(s) that affect hearing.

### Deafness and Touch

**Figure 6:**
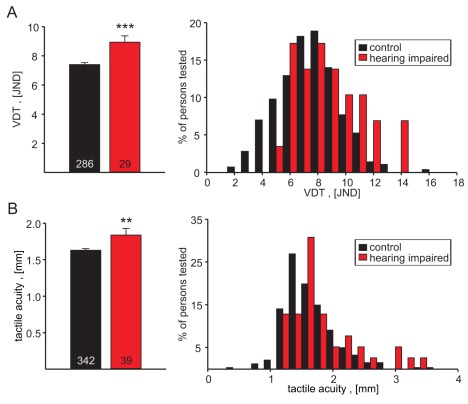
Touch sensitivity in a cohort of hearing impaired individuals compared to a cohort of normal hearing individuals. Mean performances in the mean vibration detection threshold test as well as in the tactile acuity test were poorer in the hearing impaired cohort. ** *p*<0.01; *** *p*<0.001; *t* test.

We asked if touch sensitivity might be affected in some forms of hereditary hearing loss. We tested touch sensitivity in a cohort of individuals aged between 14 and 20 years who were recruited for this study at a school for the hearing impaired. In total, 39 individuals were assessed for tactile acuity and 29 of these individuals were also tested for vibration detection threshold. All the participants suffered from severe congenital hearing impairment or hearing loss. It has been estimated that in around 70% of individuals suffering from severe hearing impairment from birth, there is an underlying genetic lesion [29]. Compared to the age-corrected control cohort, both vibration detection thresholds and tactile acuity were significantly elevated in hearing impaired individuals (Figure 6). The mean vibration detection threshold in the hearing impaired cohort was 8.93±0.44 JNDs compared to 7.40±0.13 JNDs (*p*<0.001; *t* test) in the control cohort (corresponding to stimulus amplitudes of 2.23 µm and 1.32 µm, respectively, Table S5). The mean tactile acuity was 1.84±0.09 mm in the hearing impaired cohort compared to 1.63±0.02 mm in the control cohort (*p*<0.01; *t* test). In both cases it appeared plausible from the distribution of individual values that the difference was primarily due to the presence of a subset of individuals with an exceptionally poor touch performance in the hearing impaired cohort (Figure 6). Thus of the 39 individuals tested for tactile acuity, five (13%) had very poor tactile acuity (defined as acuity >2.44 mm = mean of the control cohort plus 2 standard deviations), and of the 29 individuals who were also tested for VDT, two individuals (7%) performed poorly (defined as JND>11.8 = mean of the control cohort plus 2 standard deviations). The two individuals with high VDTs were not the same individuals as those with poor tactile acuity as defined above. It might be argued that the above differences may have resulted from the age corrections performed on the control data. However, we also compared data from hearing impaired individuals with a young sub-population of the control cohort, the mean age of which was not significantly different from the hearing impaired individuals (mean age controls 17.1 years, *n* = 141; mean age hearing impaired 16.3 years, *n* = 39). Comparing these data from the young cohort to our data from the hearing impaired individuals revealed that both measures of touch sensitivity were significantly different from each other (hearing impaired JND 8.1±0.4 compared to control JND 6.6±0.2; hearing impaired tactile acuity 1.7±0.1 compared to control tactile acuity was 1.5±0.1; Student's *t* test *p*<0.01 and *p*<0.05, respectively).

### Usher Syndrome and Touch

**Figure 7:**
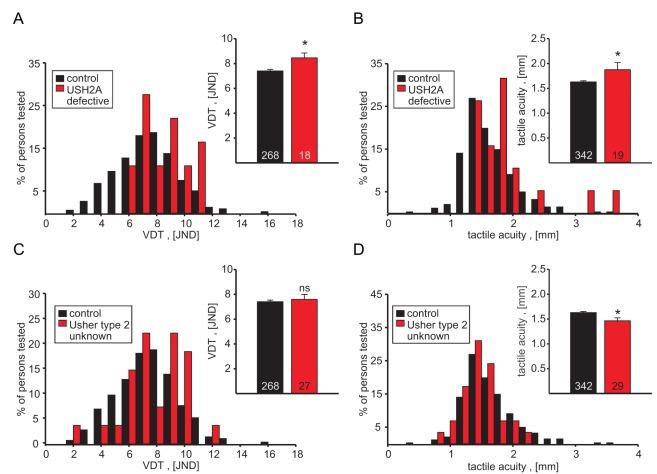
Touch sensitivity in different cohorts of people suffering from the Usher syndrome. The mean tactile acuity and vibration detection thresholds were elevated in the group of people carrying pathogenic mutations in the gene USH2A (A, B) but not in a cohort of Usher syndrome type II patients with unknown genotype where tactile acuity thresholds were lower than in the control cohort (D), and vibration detection thresholds were not significantly different (C). ^ns^, not significant; * *p*<0.05 Student's *t* test.

Our data so far strongly suggested that genetic factors that influence hearing may also influence touch. Ideally one would like to identify such genes by measuring touch performance in patients with single gene mutations that cause deafness and decrease touch sensitivity. We decided to recruit patients with Usher syndrome to participate in our study. Usher syndrome is characterized by early onset deafness with late onset retinitis pigmentosa leading to tunnel vision and blindness and can be classified into three clinical sub-types, with type 1 (USH1) typically being the most severe and type 3 (USH3) the least severe. There are nine known Usher genes, mutations in which cause the disease [30]; interestingly all known Usher gene products have been localized to the stereocilia of inner ear hair cells where mechanoelectric transduction takes place [31]. Around 60% of Usher patients suffer from the type 2 syndrome in which hearing loss is comparatively mild, with retinitis pigmentosa onset normally in the second decade. We examined patients from two cohorts of Usher patients for touch sensitivity; one cohort was obtained as part of a special consultation for Usher patients from all over Germany at the Audiology and Phoniatrics Clinic of the Charité, and the second cohort was recruited from a genotyped registry of patients diagnosed with Usher syndrome in Valencia, Spain [32]–[34]. In most cases individuals were genotyped using a microarray-based chip for the Usher genes [35]. Often this led to the identification of one mutated allele, but not the second allele (see Table S4). Thus the data presented here are derived from individuals with compound heterozygous and homozygous pathogenic *USH2A* mutations (*n* = 18), individuals with only one identified *USH2A* mutant allele (*n* = 18), as well as individuals with clinically diagnosed Usher syndrome type 2 in which no genotype has been determined (*n* = 29). Interestingly, we observed that the mean tactile acuity threshold was significantly elevated in patients proven to carry compound heterozygous or homozygous pathogenic mutations in the *USHA* gene; thus tactile acuity was 1.88±0.14 mm (*n* = 19) compared to 1.63±0.02 mm for controls (*p*<0.05; Student's *t* test) (Figure 7B). In addition the mean vibration detection threshold in those patients tested (*n* = 17) was also significantly elevated, (8.50±0.40 compared to 7.40±0.13 JNDs in the control cohort; Figure 7A). Interestingly, in patients with a clinically diagnosed Usher syndrome type 2, for which the underlying mutation was unknown (*n* = 26), we found no evidence of impaired vibration sensitivity (Figure 7C). This Usher syndrome type 2 cohort did not display impaired tactile acuity but rather performed significantly better than controls in the tactile acuity task (Figure 7D). We analyzed tactile acuity in all individuals with two or just one genotyped *USH2A* mutant allele (*n* = 36) and found that tactile acuity in this mixed cohort was slightly attenuated (acuity = 1.76±0. 10 mm, but this did not reach the criterion for statistical significance when compared to controls, *p* = 0.089).

In Usher patients with compound heterozygous or homozygous pathogenic *USH2A* mutations (*n* = 15), we also routinely tested temperature sensitivity traits. We found that warmth and cold detection thresholds as well were not significantly altered in these patients compared to the control cohort (Figure S7). Heat pain threshold was slightly but significantly altered so that patients with compound heterozygous or homozygous pathogenic *USH2A* mutations had heat pain thresholds of 43.6±0.7°C compared to 44.9±0.2 in the control cohort (*p*<0.05, Student's *t* test). When we examined temperature traits in patients with two or just one genotyped *USH2A* mutant allele (*n* = 32), we noted that this cohort performed significantly better in the warmth detection task (warmth detection threshold 0.94±0.09 Δ°C compared to 1.31±0.05 Δ°C in controls, *p*<0.01); all other temperature traits did not differ from controls. It should be noted that the effect on warmth detection was only apparent in a mixed cohort in which the second mutation was not always known; thus mutations in genes other than *USH2A* might conceivably cause this effect. Usher type 2 patients for which the underlying mutation was not known showed normal performance in the temperature detection task (unpublished data).

### Touch and Blindness

**Figure 8:**
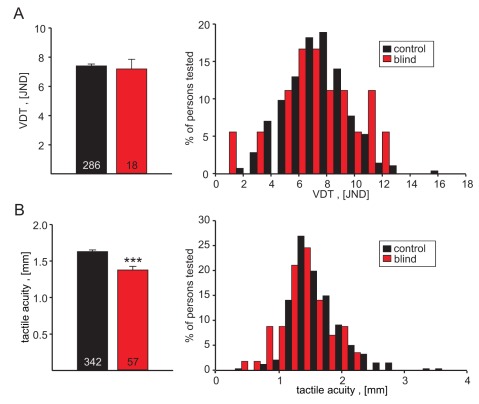
Touch sensitivity in a cohort of Braille reading blind people. The vibration detection threshold was not different in comparison to a control cohort, but the tactile acuity was significantly higher in the blind cohort. *** *p*<0.001; *t* test.

It is often assumed that the loss of one sensory modality is associated with a learned increase in the acuity of other sensory modalities. Wecarried out a study to assess the effects of blindness per se on touch sensitivity as assessed by measuring tactile acuity and vibration detection threshold. The cohort studied here (*n* = 57) was recruited at an occupational training center for blind people and the cause of blindness was sometimes presumed to be genetic but in many individuals had been caused by accidents or other non-genetic causes. The severity of visual impairment varied in the tested individuals, but was in all cases so severe that the test persons were using the Braille system to read. In agreement with previous studies of tactile acuity we found that the mean acuity of the index finger was significantly better in the blind group [36]–[38]; mean tactile acuity was 1.38±0.05 mm compared to 1.63±0.02 in the control cohort (*p*<0.001, *t* test) (Figure 8B). In the same blind cohort, vibration detection threshold was not found to be different compared to the control cohort (Figure 8A). The enhanced acuity observed in blind patients was not limited to the preferred reading finger as measurements from the contralateral finger showed that the mean acuity was not significantly different from that of the main Braille reading finger (acuity of the main Braille reading finger was 1.18±0.06 mm compared to 1.26 mm±0.08 in the contralateral index finger). These results are in broad agreement with the view that tactile acuity can be improved, possibly through learning mechanisms involving cortical plasticity. Importantly, for the present study the results show that vibration detection thresholds are probably not sensitive to such learning effects.

## Discussion

We show here using two psychophysical measures that touch performance varies considerably in the normal human population. Importantly, we could show in a classical twin study that a large part of touch performance variability (between 27% and 52%) can be accounted for by genetic factors. We also confirmed and extended published work showing that human performance traits that depend on mechanosensory systems (e.g. hearing and baroreflex sensitivity) are also partly genetically determined [39]–[43]. We have gone one step further and provide evidence that there are common genetic determinants that influence both hearing and touch. We provide three key sets of experimental data to support this conclusion. First, we show that in a normal human population there is a significant correlation between touch and hearing performance, put simply if you have good hearing there is a higher chance that you will have good touch acuity. These so called intermodal correlations were fascinating but do not establish a causal link between hearing and touch. To this end we next examined a cohort of individuals with congenital hearing impairment. A high proportion of these hearing impaired young adults displayed very poor touch performance. In the third set of experiments with a cohort of Usher syndrome patients, we identified a single gene, *USH2A* or *Usherin*, mutations in which are associated with poor touch acuity as well as congenital hearing loss and adult onset blindness. Patients with Usher syndrome in which the underlying mutation was not known did not show reduced touch performance. As well as identifying a gene that influences both hearing and touch acuity, we can conclude that there is probably a larger set of as yet unidentified genes that influence both touch and hearing. The functional characterization of such genes will help in the molecular characterization of normal and abnormal touch sensation.

Classical twin studies are used to estimate the heritable component that contributes to phenotypic variation in complex traits. We show that two measures of touch sensation, tactile acuity (*h^2^* = 0.27) and vibration detection threshold (*h^2^* = 0.52), are heritable (Figure 1). This finding is important as it allowed us to ask the question of whether common genetic factors may affect hearing and touch (see below). As part of our twin study we also measured temperature sensation. A striking feature of the cutaneous sensory system in humans is the capacity to perceive tiny increases (warming) and decreases (cooling) of the skin; thus changes of a fraction of a degree can be perceived when a surface area of ∼9 cm^2^ is warmed or cooled (Figure 4). The heritability of temperature sensation has to our knowledge not been examined, and we find significant heritability both for warmth and cold detection thresholds (*h^2^* = 0.37 and *h^2^* = 0.40, respectively).

Twin studies have previously been used to show that hearing acuity is highly heritable in middle-aged and elderly persons [41],[42],[44]; we have extended these finding to confirm the heritability of hearing acuity in a much younger cohort of twins than previously studied (average age 29 years). Hearing acuity is likely influenced by genetic factors that can act at any point along the auditory pathway; therefore, we and others have also measured evoked otoacoustic emissions (EOAE), which is a more direct measure of the mechanosensory function of outer hair cells [45]. High heritability values have already been reported for EOAE [46], but it is notable that with our larger twin sample heritability estimates were exceptionally high (e.g., EOAE strength *h^2^* = 0.80–0.93 compared to the 0.65–0.85 reported by McFadden [46]) (Figure 2). The activity of the baroreflex in resting participants has also been shown to be heritable in a twin study [43], and we could confirm this here in an independent cohort (Figure 3). Heat pain thresholds have been reported to be heritable in a twin study [14]; however, in our cohort we could not detect significant heritability of this parameter (Figure 4). In contrast to the studies of Norbury and colleagues, which exclusively recruited female twins [14], we recruited both males and females, which may have made it difficult for us to detect a heritability that is perhaps more robust in females.

The starting hypothesis of this study was that common genetic factors may influence hearing and touch. One strong hint that this may be so is that intermodal mechanosensory correlations were almost as strong as the expected intramodal correlations between traits. Thus statistically significant correlations were found between hearing acuity and tactile acuity, EOAE reproducibility and tactile acuity, and EOAE strength and baroreflex sequence frequency (Figure 5). These findings suggested, but do not prove, that gene variants may have an influence on more than one mechanosensory modality. We followed up on these results by asking if individuals with congenital deafness have altered cutaneous sensation. Strikingly, we found that hearing impaired individuals perform on average very poorly compared to controls in both touch tasks used here, and this was highly significant (Figure 6). It is likely that this effect is due to a subpopulation of individuals in this cohort who have very poor touch performance. Thus when one takes all individuals together who perform poorly (defined as > control mean + 2 SD) in both the acuity task and the vibration detection test, then up to 20% of the cohort could be considered to have a touch impairment. The cohort chosen was a random sample of hearing impaired individuals and thus probably represents a wide range of affected genetic loci that cause deafness. It follows that the surprisingly high proportion of individuals with poor touch performance is unlikely to be due to the influence of just one deafness gene. An alternative explanation is that the lack of auditory nerve activity in these individuals negatively affects the development of the somatosensory system. Interplay between touch processing and auditory processing has been reported; for example, the auditory cortex can be activated by tactile stimuli [47],[48]. It seems unlikely that lack of auditory input would adversely affect processing of touch-related sensory information, since it has been shown that the auditory cortex is even more active in response to tactile stimuli in deaf test persons compared to normal hearing individuals [24]. Our finding that most individuals with severe hearing impairment do not have altered touch sensitivity shows that auditory impairment per se does not necessarily negatively impact touch.

How could hearing genes influence touch sensitivity? Common features of congenital hearing loss range from disorganization of the stereocilia, where mechanotransduction takes place, to complete degeneration and loss of sensory hair cells [1]–[3]. There is, however, no indication that somatic sensory neurons require cilia for their function and so loss of hearing gene function could act at many other levels. In the case of late onset deafness caused by dominant negative mutations in the gene encoding the potassium ion channel protein KCNQ4 (DFNA2-type monogenic hearing loss), hair cells degenerate apparently due to sustained depolarization [49]. We have recently shown that the KCNQ4 protein has a specialized role in the transformation of receptor potentials into action potentials in specific types of mechanoreceptors [50]. Furthermore, loss of KCNQ4 function is associated with better vibration detection threshold performance, but only at low vibration frequencies [50]. Some hearing genes have also been shown to affect synapses made between the hair cell and the sensory afferents that convey the auditory signals to the brain [51],[52]. It is thus quite conceivable that deafness genes that influence synaptic properties may also have consequences for the functional properties of sensory neuron synapses in the somatosensory system.

The effect of sensory loss on touch sensitivity has been studied before in the case of blindness. Despite the common notion that blind people are superior in tactile tasks, the picture arising from previous studies is not as clear. A number of different tests have been employed to address this question, with some producing better results in blind cohorts and some not [37],[53]–[55]. This was also the case in our blind cohort; thus we found vibration detection thresholds unchanged in a blind cohort, but the tactile acuity clearly enhanced (Figure 7). Tactile acuity has previously been tested in blind participants with conflicting results [36],[37],[53]; however, blindness has never been associated with reduced touch sensitivity.

We wished to identify single genes that influence hearing and touch and thus decided to study individuals with Usher syndrome. Usher syndrome is very well characterized at the genetic level, with many alleles known to affect at least 10 genes including *MYO7A* (MIM:276903), *USH1C* (MIM:605242), *CDH23* (MIM:605516), *PCDH15*(MIM: 605514), *SANS* (MIM: 607696), *USH2A*, *VLGR1*(MIM: 602851), *WRHN* (MIM: 607928), *USH3A*(MIM: 606397), *and PDZD7*(MIM: 612971) [2],[56],[57]. Interestingly, all the Usher genes characterized in detail so far are expressed in sensory hair cells, and in several cases the protein product has even localized to sites of transduction at the tips of the stereocilia [2]. Indeed, there is solid evidence showing that the tip-link, which is necessary for transferring force to open mechanotransduction channels, is made up of two Usher gene protein products, cadherin-23 and protocadherin-15 [58],[59]. In DRG neurons we have recently obtained evidence that a very large extracellular protein tether (∼100 nm in length) is required for the gating of mechanosensitive currents found in touch receptors [60],[61]; the identity of this tether protein is not known, but its biochemical properties do not match that of tip link proteins cadherin-23 and protocadherin-15 [60]. Here we show that elevated tactile acuity and vibration detection thresholds were observed in patients suffering from Usher syndrome type II caused by mutations in *USH2A* (Figure 7A). USH2A is a transmembrane protein with a very large extracellular domain, in principle long enough to extend 100 nm into the extracellular space [62]. In hair cells the USH2A protein is localized at the base of the stereocilia and is thought to be part of the ankle links that connect adjacent stereocilia [63],[64]. USH2A protein could be detected only in the developing cochlear hair cells, but was also detected at later stages in vestibular hair cells. USH2A has been shown to bind to other Usher proteins [63],[65] as well as to collagen IV [66] and could be a link between the inner network of Usher proteins and the extracellular matrix. Stereocilia bundles are disorganized in mice with a targeted deletion of the *USH2A* gene, and these mice are also deaf [67]. The biochemical properties of the USH2A protein make it a conceivable candidate for the tether visualized in sensory neurons.

Human *USH2A* is an extraordinarily large gene consisting of 72 exons, which can encode a protein with a length of 5,222 amino acids [62]. Several different transcripts have been identified for the *USH2A* gene and so it is conceivable that mutations in this gene may have differential effects on protein products expressed in different tissues (e.g., hair cells versus sensory neurons). Most patients in which one pathogenic *USH2A* mutation has been identified are probably also carriers of a second mutation in the same gene [68]. Consistent with this prediction we observed that a mixed cohort of patients homozygous or heterozygous for pathogenic *USH2A* mutations also exhibited impaired touch sensitivity, although this difference was not statistically significant. Interestingly, many individuals with Usher syndrome type II in our study did not exhibit impaired touch acuity, although previous analyses of large populations of these patients have provided estimates that the majority (up to 70%) may carry pathogenic mutations in the *USH2A* gene [33],[69],[70]. Interestingly, Usher type 2 patients for which no mutation in the *USH2A* was known performed on average better in the touch acuity test than controls (Figure 7D). These patients also suffered from visual impairment, and it is possible that they, like blind people, had learned to improve their tactile acuity. Mutation in two other genes, *WHRN* and *VLGR1*, can lead to Usher syndrome type II [71],[72], but we could not confirm any such cases in our cohort. The newly demonstrated effect of *USH2A* gene mutations on touch acuity shown here clearly warrants a detailed study of this protein in the somatosensory system.

In summary, our study demonstrates that human touch sensitivity is indeed accessible at a genetic level and we provide evidence for shared genetic factors influencing different mechanosensory systems, especially touch and hearing. It is in fact quite likely that the identification of single gene mutations that affect touch may provide a wealth of new insight into genes that determine the development, connectivity, as well as the nature of mechanosensory transduction in the touch system.

## Material and Methods

All experiments performed on human participants were approved by the local ethic committees. Each study participant was asked to complete a statement of informed consent.

### Twin Study Testing Procedure

With few exceptions the testing procedure was as follows: Twins were tested starting in the morning in a quiet room. The setting was usually a hospital examination room that was centrally heated with regular air exchange via a centralized ventilation system. Twins took turns being tested for baroreflex sensitivity and audiometry assessments. The twins were subsequently tested for tactile acuity, vibration detection threshold, and temperature sensitivity (in the order: cold detection, warmth detection, heat pain, and cold pain thresholds). Finally blood samples were taken. Audiometry testing was carried out by trained personnel of the Clinic for Audiology and Phoniatrics of the Charité–Berlin. All other testing was carried out by the same investigator. The entire testing procedure for one twin pair typically lasted about 4 h.

### Vibration Detection Threshold

Vibration detection thresholds were determined using the CASE IV system (WR Medical Electronics) [73]. In the vibration detection test a transformed-rule up and down method was applied [74] in connection with a two-interval forced choice test. A vibration stimulator was applied below the nail of the little finger. To prevent possible auditory detection of the vibration stimulator, (normal hearing) test persons wore headphones, which produced a low, continuous tone during the test. A sinusoidal 125 Hz vibration stimulus with duration of 1.68 s was applied during one of two periods indicated to the test persons. The participants then chose the period (indicated by a 1 or 2) during which they thought the vibration had been applied. A step towards the next smallest amplitude was made when the test persons responded correctly to six times in a maximum of eight trials; otherwise a step to the next largest amplitude was made. Eight such reversal points were determined. The calculated vibration detection threshold corresponded to the vibration amplitude at which approximately 75% of the answers are correct [67]. The amplitude magnitude steps were just noticeable differences (JNDs) that have been previously determined and roughly resemble a logarithmic representation of the amplitude in µm (Table S5).

### Tactile Acuity Test

Tactile acuity was determined with a two interval forced choice grating orientation determination test using the Tactile Acuity Cube. In the tactile acuity test a transformed-rule up and down method was applied [74]. Test persons placed their hand, with the palmar surface facing upward, on a table and (sighted) test persons were blindfolded. The Tactile Acuity Cube was applied for 1 s to the fingerpad, in a way that the cube exerts its whole weight on the finger (233 g). Test persons had to determine if the orientation of the gratings on the cube was parallel or perpendicular to the fingers, starting with the widest grating width. Each grating width was tested two times and if two answers were correct, the next, smaller width was tested; this was continued until the test person answered incorrectly. The grating width was then increased stepwise again until the two orientations of a width were determined correctly again. Thirteen of these reversal points were determined and the mean of the last 10 taken as the threshold. The threshold corresponds to the grating width where the probability of a correct answer is 0.707. Thresholds were determined for the little finger and the index finger and the mean of this threshold taken as the tactile acuity [74]. In each case the participants were asked which their preferred hand was and this hand was used for the tactile acuity measurement.

### Audiometry

Audiometry was carried out in the Klinik für Audiologie und Phoniatrie, Charité–Universitätsmedizin Berlin employing the standard procedures for clinical use. For hearing acuity the pure tone thresholds in decibels (dB, Sound pressure level, SPL) at 0.5, 1, 2, and 4 kHz were determined using a ST36 Audiometer (Maico) and the mean calculated. The otoacoustic emissions were measured using an OAE (Otodynamics). Otoacoustic emissions were evoked by 1 ms clicks spanning a frequency range from 0–6 kHz. The measured parameters were the reproducibility of the frequency distribution of consecutively evoked emissions in percentages and the overall intensity of the emissions in dB (SPL).

### Baroreflex Measurement

Studies were conducted with the participant in a supine body position. Five-minute recordings were obtained after 10 min of rest. Blood pressure (BP) was measured in the left arm by automated oscillometric device (Dinamap) as well as continuously by Finapres (Ohmeda) BP monitor attached to the middle finger of the right hand. The participant's right hand was kept at heart level. ECG was recorded continuously. Data were analog-digital converted (both channels at 1 kHz), peak detection (R peak, systolic BP, and diastolic BP), and subsequent analyses were done using the PV-wave software (VisualNumerics). Sequences of at least three coupled minimum steps of 0.5 mmHg BP changes and 5 ms RR–interval changes with minimum correlation coefficients of 0.85 were detected and their slopes taken as the baroreflex sensitivity in ms/mmHg. Blood pressure levels were allowed for by regression for both baroreflex sequence slope and frequency before analysis of heritability or phenotypic correlations.

### Temperature Sensitivity Tests

Temperature sensitivity was determined using the TSA-II System (Medoc advanced medical systems) according to the manufacturer's instructions. The thresholds were determined using the ascending method of limits. A peltier thermode (3 cm×3 cm) was placed in the middle of the volar forearm. The baseline temperature for all four tests was 32°C and the temperature change rate was 0.5°C/s. In the temperature change detection tests, the test persons indicated when they felt a change in skin temperature. For warming and cooling ramps, the mean of four thresholds was calculated. In the temperature pain threshold tests, the test persons indicated when a rising or falling temperature became painful. Here the mean of three thresholds was calculated.

### Heritability and Statistical Analysis

Heritability (*h^2^*) estimates were calculated by structural equation modeling using the Mx software [27]. At first, heritabilities were estimated in an ACE model, in which the variation of a trait is composed of the variations of additive genetic effects (A), shared environment effects (C), and unique environment effects (E); accordingly the co-variation of a trait between monozygotic twins is equivalent to the variation of A and E, whereas it is 0.5 times the variation of A and the variation of E in dizygotic twins. Subsequently, AE and CE sub-models were tested and the best fitting model selected according to the AIC (Akaikes information criterion). For the selected model the heritability (proportion of A effects) was estimated. An age correction was performed before the genetic analysis in analogy to the age correction outlined in Table S1 using the data of the twins only. Transformation of datasets was conducted, if necessary, so that a normality test was passed (Kolgomorov-Smirnov test).

Zygosity was tested using 9,080 informative autosomal SNPs from a custom designed single nucleotide polymorphism (SNP) Immunochip array [25],[26]. Informative SNPs for zygosity testing were selected on the basis of the following criteria; there was a minimal distance of 100,000 base pairs between SNPs, the SNPs had minimal minor allele frequencies (>0.1), redundant SNPs were excluded (i.e., those in linkage disequilibrium), and SNPs on the X-chromosome were excluded. The data were analyzed and genotype calls made using the Illumina Genome studio software [75]. Standard statistical analysis was done using GraphPad Prism software, calculation of false discovery rates was done using the QVALUE software written by David Siegmund and John Storey [28].

## Supporting Information

Figure S1 Age dependence of touch sensitivity traits. The vibration detection threshold (A) and the tactile acuity (B) showed strong age dependence, with a lower sensitivity in older participants. Solid lines are regression lines. Equations of the regressions are listed in Table S1.
(EPS)

Figure S2 Age dependence of hearing traits. Performance in all three traits deteriorates with increasing age, especially hearing acuity (A). Solid lines are regression lines. Equations of the regressions are listed in Table S1.
(EPS)

Figure S3 Age dependence of baroreflex traits. The baroreflex sequence slopes showed a strong age-dependence decrease (A), whereas the baroreflex frequency seems to be unaffected by age (B). Solid lines are regression lines. Equations of the regressions are listed in Table S1.
(EPS)

Figure S4 Age dependence of temperature sensitivity traits. All temperature traits showed age dependence, with lower sensitivity in older participants. Solid lines are regression lines. Equations of the regression are listed in Table S1.
(EPS)

Figure S5 Sex comparison of sensory traits. Tactile acuity, otoacoustic emission reproducibility and strength, baroreflex sequence frequency, as well as the cold and warmth detection thresholds showed a significant difference in the sex comparison (B,D,E,G,H,I), whereas the other traits did not. Sensitivity or parameter magnitude was higher in female participants in all cases. ^ns^, not significant; * *p*<0.05; ** *p*<0.01; *** *p*<0.001.
(EPS)

Figure S6 Cross-correlations between the investigated sensory traits with sex-dependent values adjusted for sex. Only one intermodal significant intermodal correlation between tactile acuity and hearing acuity remains after sex correction. ^ns^, not significant * *p*<0.05; ** *p*<0.01; *** *p*<0.001. False discovery rates for *p* value cutoff at 0.05 for (1) intramodal correlations: 0.004 and (2) mechanosensory intermodal correlations: 0.28. Values were corrected for age before analysis.
(EPS)

Figure S7 Temperature sensitivity of patients suffering from Usher syndrome type II with compound heterozygous or homozygous pathogenic *USH2A* mutations (USH2A defective, *n* = 15). Mean cold and warmth detection thresholds were not significantly different in the USH2A defective cohort compared to controls, but heat pain thresholds were significantly lower in this cohort. ^ns^, not significant, * *p*<0.05.
(EPS)

Table S1 Trait–age correlations and regressions used for age correction.
(PDF)

Table S2 Sex comparison of sensory traits.
(PDF)

Table S3 Summary of cross-twin correlations and heritability estimates of investigated sensory traits.
(PDF)

Table S4 Individual mutations in the *USH2A* gene of the people tested for touch sensitivity and the corresponding tactile acuity thresholds.
(PDF)

Table S5 Conversion table for just noticeable difference to peak-to-peak amplitude in µm.
(PDF)

## Abbreviations

ACE model: model including additive genetic component, common and unique environment components
AE model: model including additive genetic component and unique environment component
BP: blood pressure
CE model: model including common and unique environment components
CI: confidence interval
DRG: dorsal root ganglion
EOAE: evoked otoacoustic emissions
FDR: false discovery rate
*h*^2^: heritability
JND: just noticeable differences
*r*: correlation coefficient
SD: standard deviation
SNP: single nucleotide polymorphism
VDT: vibration detection threshold
