# Supplementary material for: A Genetic Basis for Mechanosensory Traits in Humans
Source: PLoS Biol. 2012 May 1;10(5):e1001318. doi: 10.1371/journal.pbio.1001318 (PMC3341339; doi:10.1371/journal.pbio.1001318)
Supplement: Table S1 — Trait–age correlations and regressions used for age correction. (PDF) [file pbio.1001318.s008.pdf]

Table S1: Trait – age correlations and regressions used for age correction.

Trait	Correlation to age (r)	Type of fit for age correction*	Coefficient of determination (R^2)	Equation
**Vibration detection threshold [JND]**	0.46, $p < 0.001$	Polynomial 2nd order	0.22	$y = 6.19 + 0.00137 \cdot x^2$
**Tactile acuity [mm]**	0.42, $p < 0.001$	Polynomial 2nd order	0.21	$y = 1.41 + 0.00025 \cdot x^2$
**Hearing acuity [dB]**	0.42, $p < 0.001$	Polynomial 2nd order	0.18	$y = 6.89 + 0.00201 \cdot x^2$
**EOAE reproducibility [%]**	0.15, $p < 0.05$	Linear	0.02	$y = 89.10 - 0.15205 \cdot x$
**EOAE strength [dB]**	0.20, $p < 0.01$	Linear	0.04	$y = 19.10 - 0.08390 \cdot x$
**Baroreflex sequence slope [ms / mmHg]**	-0.38, $p < 0.001$	Exponential decay	0.16	$y = 51.68 \cdot \exp(-0.03231 \cdot x) + 3.246$
**Baroreflex sequence frequency [1 / 5 min]**	0.01, n.s.	Linear	0.02	$y = 25.62 + 0.01067 \cdot x$
**Cold detection threshold [°C]**	-0.41, $p < 0.001$	Polynomial 2nd order	0.22	$y = 31.51 - 0.00022 \cdot x^2$
**Warmth detection threshold [°C]**	0.29, $p < 0.001$	Polynomial 2nd order	0.08	$y = 33.10 + 0.00024 \cdot x^2$
**Heat pain threshold [°C]**	0.18, $p < 0.05$	Linear	0.03	$y = 43.80 + 0.03531 \cdot x$
**Cold pain threshold [°C]**	-0.15, $p < 0.05$	Polynomial 2nd order	0.02	$y = 15.37 - 0.00184 \cdot x^2$

n.s. – not significant ; *chosen from a set of equations (linear, polynomial 2nd order, exponential, exponential association, exponential decay) according to Akaike's Information Criterion (AIC); y – trait parameter; x – age.
