# Supplementary material for: A Genetic Basis for Mechanosensory Traits in Humans
Source: PLoS Biol. 2012 May 1;10(5):e1001318. doi: 10.1371/journal.pbio.1001318 (PMC3341339; doi:10.1371/journal.pbio.1001318)
Supplement: Table S2 — Sex comparison of sensory traits. (PDF) [file pbio.1001318.s009.pdf]

Table S2: Gender comparison of sensory traits

Trait	Female	Male	Two tailed t-test
**Vibration detection threshold [JND]**	7.22 ± 0.10; n = 170	7.59 ± 0.26; n = 103	$p > 0.05$; ns
**Tactile acuity [mm]**	1.54 ± 0.03; n = 189	1.68 ± 0.04; n = 140	$p < 0.01$; **
**Hearing acuity [dB]**	8.32 ± 0.35; n = 94	9.29 ± 0.44; n = 60	$p > 0.05$; ns
**EOAE reproducibility [%]**	86.83 ± 0.86; n = 123	82.46 ± 1.14; n = 71	$p < 0.01$; **
**EOAE strength [dB]**	17.9 ± 0.34; n = 122	15.36 ± 0.49; n = 71	$p < 0.001$; ***
**Baroreflex sequence slope [ms / mmHg]**	24.21 ± 1.30; n = 113	23.82 ± 1.76; n = 60	$p > 0.05$; ns
**Baroreflex sequence frequency [1 / 5 min]**	27.51 ± 1.36; n = 113	21.72 ± 1.33; n = 60	$p < 0.01$; **
**Cold detection threshold [°C]**	-0.66 ± 0.04; n = 103	-0.8 ± 0.06; n = 69	$p < 0.05$; *
**Warmth detection threshold [°C]**	1.22 ± 0.06; n = 99	1.61 ± 0.10; n = 70	$p < 0.01$; **
**Heat pain threshold [°C]**	44.79 ± 0.20; n = 99	45.23 ± 0.26; n = 70	$p > 0.05$; ns
**Cold pain threshold [°C]**	13.77 ± 1.00; n = 108	12.7 ± 1.20; n = 68	$p > 0.05$; ns

Listed are the values ± SEM. ns - not significant; * - $p < 0.05$; ** - $p < 0.005$; *** - $p < 0.001$; t-test.
