# Supplementary material for: A Genetic Basis for Mechanosensory Traits in Humans
Source: PLoS Biol. 2012 May 1;10(5):e1001318. doi: 10.1371/journal.pbio.1001318 (PMC3341339; doi:10.1371/journal.pbio.1001318)
Supplement: Table S3 — Summary of cross-twin correlations and heritability estimates of investigated sensory traits. (PDF) [file pbio.1001318.s010.pdf]

Table S3: Summary of Cross-twin correlations and heritability estimates of sensory traits

**Trait**	**r MZ**	**r DZ**	h^2	**95% CI**	**Model**
**Vibration detection threshold [JND]**	0.53	0.19	0.52	0.33 - 0.67	AE
**Tactile acuity [mm]**	0.28	0.10	0.27	0.05 - 0.46	AE
**Hearing acuity [dB]**	0.74	0.35	0.8	0.67 - 0.87	AE
**EOAE reproducibility [%]**	0.79	0.18	0.76	0.62 - 0.85	AE
**EOAE strength [dB]**	0.89	0.36	0.88	0.80 – 0.93	AE
**Baroreflex sequence slope [ms / mmHg]**	0.39	0.26	0.39	0.17 - 0.57	AE
**Baroreflex sequence frequency [1 / 5 min]**	0.57	0.00	0.56	0.34 - 0.71	AE
**Cold detection threshold [°C]**	0.41	-0.02	0.4	0.16 – 0.60	AE
**Warmth detection threshold [°C]**	0.44	0.11	0.37	0.14 – 0.56	AE
**Heat pain threshold [°C]**	0.25	0.31	-	-	CE
**Cold pain threshold [°C]**	0.45	0.39	-	-	CE

*r*MZ, *r*DZ = intraclass correlation of monozygotic and dizygotic twin pairs; *h2* = heritability estimate; 95% CI = 95% confidence interval; model = best fitting model; AE = model with genetic and unique environment component; CE = model with common environment and unique environment component.
