# Supplementary material for: A Genetic Basis for Mechanosensory Traits in Humans
Source: PLoS Biol. 2012 May 1;10(5):e1001318. doi: 10.1371/journal.pbio.1001318 (PMC3341339; doi:10.1371/journal.pbio.1001318)
Supplement: Table S4 — Individual mutations in the USH2A gene of the people tested for touch sensitivity and the corresponding tactile acuity thresholds. (PDF) [file pbio.1001318.s011.pdf]

Table S4: Individual mutations in the *USH2A* gene of the people tested for touch sensitivity and the corresponding tactile acuity thresholds.

n	Mutation 1		Mutation 2		tactile acuity [mm]
**1**	c.2299delG	p.E767SfsX21	c.4381C>T	p.Q1461X	1.34
**1**	c.2299delG	p.E767SfsX21	c.11864G>A	p.W3955X	3.23
**5**	c.2299delG	p.E767SfsX21	c.2299delG	p.E767SfsX21	1.50, 1.80, 1.99, 1.33 1.72
**1**	c.1724G>A	p.C575Y	c.11864G>A	p.W3955X	1.65
**1**	c.2135delC	p.S712X	c.2431_2432delAA	p.K811DfsX11	3.66
**1**	c.2431_2432delAA	p.K811DfsX11	c.2431_2432delAA	p.K811DfsX11	2.06
**1**	c.1214delA	p.N405IfsX3	c.4029T>A or G	p.N1343K	1.74
**2**	c.2299delG	p.E767SfsX21	c.11234dupA	p.Y3745X	1.81, 1,83
**1**	c.2299delG	p.E767SfsX21	c.1256G>T	p.C419F	1.81
**1**	c.653T>A	p.V218E	5777+1G>A	splice site	1.37
**1**	c.4515_4518delAGAG	p.R1505SfsX7	c.13316C>T	p.4439I	1.53
**2**	c.11864G>A	p.W3955X	c.1036A>C	p.N346H	1.56, 2.37
**1**	c.9424G>T	p.G3142X	c.2299delG	p.E767SfsX21	1.34
**8**	c.2299delG	p.E767SfsX21	unknown	unknown	2.83, 2.37, 1.64, 2.19 1.27, 1.37, 1.90, 1.26
**1**	c.653T>A	p.V218E	unknown	unknown	1.11
**1**	c.1606T>C	p.C536R	unknown	unknown	1.53
**1**	c.13316C>T	p.T4439I	unknown	unknown	1.36
**5**	c.11864G>A	p.W3955X	unknown	unknown	1.22, 0.80, 1.67, 1.55, 2.19
**1**	c.486-14G>A	splice site	unknown	unknown	1.33
