# Supplementary material for: A Genetic Basis for Mechanosensory Traits in Humans
Source: PLoS Biol. 2012 May 1;10(5):e1001318. doi: 10.1371/journal.pbio.1001318 (PMC3341339; doi:10.1371/journal.pbio.1001318)
Supplement: Table S5 — Conversion table for just noticeable difference to peak-to-peak amplitude in µm. (PDF) [file pbio.1001318.s012.pdf]

Table S5: Conversion table from JND to µm for the CASEIV vibration detection threshold test

JND	µm
1	0.106
1.5	0.133
2	0.166
2.5	0.205
3	0.251
3.5	0.305
4	0.370
4.5	0.448
5	0.539
5.5	0.649
6	0.778
6.5	0.932
7	1.114
7.5	1.332
8	1.590
8.5	1.897
9	2.263
9.5	2.696
10	3.212
10.5	3.824
11	4.553
11.5	5.418
12	6.447
12.5	7.669
13	9.122
13.5	10.849
14	12.901
14.5	15.340
15	18.239
15.5	21.684
16	25.778
16.5	30.644
17	36.428
17.5	43.301
18	51.471
18.5	61.180
19	72.718
19.5	86.431
20	102.730
